# Association between Poor Glycemic Control, Impaired Sleep Quality, and Increased Arterial Thickening in Type 2 Diabetic Patients

**DOI:** 10.1371/journal.pone.0122521

**Published:** 2015-04-14

**Authors:** Koichiro Yoda, Masaaki Inaba, Kae Hamamoto, Maki Yoda, Akihiro Tsuda, Katsuhito Mori, Yasuo Imanishi, Masanori Emoto, Shinsuke Yamada

**Affiliations:** Department of Metabolism, Endocrinology and Molecular Medicine, Osaka City University Graduate School of Medicine, Osaka, Japan; Emory University, UNITED STATES

## Abstract

**Objective:**

Poor sleep quality is an independent predictor of cardiovascular events. However, little is known about the association between glycemic control and objective sleep architecture and its influence on arteriosclerosis in patients with type-2 diabetes mellitus (DM). The present study examined the association of objective sleep architecture with both glycemic control and arteriosclerosis in type-2 DM patients.

**Design:**

Cross-sectional study in vascular laboratory.

**Methods:**

The subjects were 63 type-2 DM inpatients (M/F, 32/31; age, 57.5±13.1) without taking any sleeping promoting drug and chronic kidney disease. We examined objective sleep architecture by single-channel electroencephalography and arteriosclerosis by carotid-artery intima-media thickness (CA-IMT).

**Results:**

HbA1c was associated significantly in a negative manner with REM sleep latency (interval between sleep-onset and the first REM period) (β=-0.280, p=0.033), but not with other measurements of sleep quality. REM sleep latency associated significantly in a positive manner with log delta power (the marker of deep sleep) during that period (β=0.544, p=0.001). In the model including variables univariately correlated with CA-IMT (REM sleep latency, age, DM duration, systolic blood pressure, and HbA1c) as independent variables, REM sleep latency (β=-0.232, p=0.038), but not HbA1c were significantly associated with CA-IMT. When log delta power was included in place of REM sleep latency, log delta power (β=-0.257, p=0.023) emerged as a significant factor associated with CA-IMT.

**Conclusions:**

In type-2 DM patients, poor glycemic control was independently associated with poor quality of sleep as represented by decrease of REM sleep latency which might be responsible for increased CA-IMT, a relevant marker for arterial wall thickening.

## Introduction

Patients with diabetes mellitus (DM), who exhibit accelerated arteriosclerosis, are at a higher risk of coronary heart disease (CHD), even after adjusting for established CHD risk factors such as gender, age, smoking, blood pressure, and dyslipidemia [1]. Impaired quantity and quality of sleep have been established to be involved in the development of atherosclerosis and CHD [2].

Sleep is a dynamic state with its own distinctive stages that cycle throughout the night. The succession of cycles, their component stages, and the length of each stage and cycle comprise a person’s sleep architecture [3]. Patients with type 2 DM have been reported to have higher rates of insomnia [4] and to demonstrate a significantly decreased amount of slow-wave sleep (SWS; American Academy of Sleep Medicine sleep scoring criteria [5], stages 3 and 4) [6]. We recently demonstrated that poor glycemic control significantly and independently associated with morning blood pressure surge [7]. Together with the previous finding demonstrating the close relationship between decreased SWS and increased nocturnal blood pressure [8], inadequate glycemic control might lead to the impaired quantity and quality of sleep in DM patients. Furthermore, elderly persons with insomnia exhibit elevated level of intima-media thickness at carotid artery (CA-IMT) [9], which is an early quantitative marker of generalized arteriosclerosis that is associated with incident CHD and its risk factors [10]. Taken together, these results suggest that poor glycemic control could accelerate arteriosclerosis in DM patients by causing sleep disorders.

In this study, we evaluated (i) the association of glycemic control with sleep length and quality as assessed by single-channel electro-encephalography (EEG), and (ii) the association between sleep disturbance and arteriosclerosis, as assessed by CA-IMT, in patients with type 2 DM.

## Subjects and Methods

### Subjects and design

The cross-sectional study protocol was approved by the Ethics Committee of Osaka City University Graduate School of Medicine (registration number 307–9). The subjects of this study were inpatients in our vascular laboratory at Osaka City University Hospital. Written informed consent was obtained from each patient and the study was conducted in full accordance with the Declaration of Helsinki.

Eighty consecutive patients with type 2 DM underwent single-channel EEG and CA-IMT between April 2012 and June 2014. Among them 7 were excluded because of taking any sleeping promoting drug. Then 10 patients with renal dysfunction [estimated glomerular filtration rate (eGFR) <60 ml/min per 1.73 m^2^] and/or prevalent liver disease were excluded. The data of the 63 remaining patients were used for subsequent analysis. The diagnosis of type 2 DM was made on the basis of a history of DM or according to the Japan Diabetes Society criteria [11]. BMI was calculated as body weight (kg)/[height (m)]^2^.

Ten of the patients were managed by dietary therapy alone, 19 patients were receiving insulin therapy, and 34 patients were receiving oral hypoglycemic agents [8 with sulfonylureas, 14 with biguanides, and 12 with a combination of oral hypoglycemic agents]. Furthermore, 21 patients were receiving statins and 25 patients were receiving anti-hypertensive drugs [8 receiving calcium channel blockers (CCB), 8 receiving an angiotensin-converting enzyme inhibitor (ACEI) and/or an angiotensin receptor blocker (ARB), and 9 receiving a combination of anti-hypertensive drugs].

### Measurements and analysis of EEG data

All patients underwent an overnight electroencephalogram monitoring, which was obtained using a single-channel EEG (SLEEP SCOPE; Sleep Well Co., Osaka, Japan), as previously described for sleep scoring programs [12, 13]. According to established practice [5], the recorded night was divided into 30-s sequential periods, and manually classified into rapid eye movement (REM) sleep and non-REM sleep, which is again classified into light sleep (S1/S2) or SWS. Total sleep time was calculated as the total sleep period minus the time spent awake during the sleep period. Sleep onset was defined by the first occurrence of stage 2 sleep, which was followed by 5 min of continuous sleep composed of stage 1, 2, 3, 4 or REM sleep. REM sleep latency was defined as the number of minutes of sleep between sleep onset and the occurrence of the first REM period lasting at least 2 min as reported previously [14].

As a marker of deep sleep [15], we calculated the delta power; as previously described [16], the EEG data underwent autoregressive high-pass filtering and Hanning windowing and were subsequently decomposed in 30-s periods using the fast Fourier transform into delta and other frequency bands, furthermore, we calculated power (μV^2^) for the delta frequency band.

Polysomnographic measurement of the Apnea Hypopnea Index (AHI) was performed using respiratory flow pressure, oxygen saturation and heart rate monitor system (SAS2100, Nihonkoden Co., Tokyo, Japan) [17]. Apneas were defined as a complete cessation of airflow lasting for ≥ 10 s, and hypopnea was defined as reduction of ≥ 50% in airflow from a baseline of ≥ 10 s and associated with a 3% desaturation or an arousal. The AHI was defined as the ratio of the number of episodes of apnea and hypopnea per hour of sleep.

### Measurement of carotid atherosclerosis with the intima-media thickness by ultrasounds

The CA-IMT of the common carotid artery was measured by ultrasonic diagnosis equipment with a high-resolution real-time 6-MHz linear scanner (ProSound F75; Aloka Co., Tokyo, Japan) that was programmed by the IMT measurement software, intimascope (Media Cross Co., Tokyo, Japan), as previously reported [18]. In brief, patients were examined in a supine position. Images were obtained in the 20 mm proximal to the origin of the bulb at the far wall of the left common carotid artery. Computer-based IMT was evaluated by 3-point evaluation which refers to the average value of 3-point IMT, including the point of max IMT and two surrounding points on both sides (each 10mm distant from the center of images).

### Physiological and biochemical parameters

Blood pressure was measured early in the morning in the fasted state. Blood and urine samples were obtained after an overnight fast. Fasting plasma glucose levels were measured by the glucose oxidase method. Glycated hemoglobin A1c (HbA1c) was determined by using routine HPLC and a latex agglutination immunoassay and expressed as the National Glycohemoglobin Standardization Program equivalent value [19]**.** Serum creatinine, total cholesterol, and high-density lipoprotein cholesterol (HDL-C) were measured using an autoanalyzer (Hitachi 7450; Hitachi Co., Tokyo, Japan). Non-HDL-C was calculated by subtracting HDL-C from total cholesterol. Urinary albumin was determined using turbidimetric immuneassay (TIA; Wako Co., Tokyo, Japan). eGFR, as a measure of renal function, was based on the equation proposed by the Japanese Society of Nephrology [20]: eGFR (milliliters per min per 1.73 m2) = 175 ×serum creatinine^−1.154^ × age^−0.203^ × F (where F = 1 if male, and 0.742 if female).

### Statistical analysis

Continuous variables with normal distribution were expressed as the mean (SD). The median (interquartile range) was used for the DM duration, CA-IMT, SWS, delta power, and AHI because of their skewed distribution. Unpaired samples were non-parametrically analyzed using the Mann-Whitney *U* test. Correlation coefficients were calculated by simple and multiple regression analysis after logarithmic transformation of the delta power because this transform best led to distributions that approximated the normal distribution [21]. Univariate regression analysis was performed using non-parametric Spearman rank correlation test. Patients were grouped into 3 categories of glycemic control: HbA1c < 7% (53 mmol/mol) desirable, 7–9% (53–75 mmol/mol) suboptimal, and ≥9% (75 mmol/mol) poor based on literature [22]. A separate analysis of variance (ANOVA) was calculated for each EEG date to determine differences among the 3 groups. Post hoc analysis was conducted by Dunnett's significant difference. P-values < 0.05 were considered statistically significant. All data were analyzed using Stat View version 5.0 J (Abacus Concepts, Incorporated, Berkeley, California).

## Results

### Clinical and biochemical profiles of the patients

In total, 63 patients with type 2 DM were enrolled and their clinical and biochemical profiles are summarized in Table 1. The mean age was 57.5 years (SD, 13.1). Thirty two patients (51%) were male and 31 (49%) were female. The median duration of DM was 7.0 years (2.0–16.0 years). The number of current smoker and diabetic painful neuropathy were 17 patients (27%) and 9 patients (14%), respectively. The mean morning systolic blood pressure was within the normal range. The mean levels of non-HDL-C, eGFR and urine albumin-to-creatinine ratio were within the normal range, respectively. CA-IMT did not differ significantly between those with and without insulin and/or oral hypoglycemic agents (0.68 (SD, 0.22) vs. 0.58 (SD, 0.13) mm, p = 0.172), statins (0.68 (SD, 0.24) vs. 0.66 (SD, 0.20) mm, p = 0.912), and anti-hypertensive drugs (0.65 (SD, 0.22) vs. 0.67 (SD, 0.21) mm, p = 0.704). No significant differences were found in CA-IMT between current smokers and non-smokers (0.67 (SD, 0.30) vs. 0.66 (SD, 0.18) mm, p = 0.520).

**Table 1:** Clinical and biochemical profiles of patients.

Measure	N = 63
Gender [Male/Female]	32/31
Age (years)	57.5 (13.1)
DM duration (years)	7.0 [2.0–16.0]
Current smoker, n (%)	17 (27)
Diabetic painful neuropathy, n (%)	9 (14)
BMI (Kg/m^2^)	26.0 (5.7)
Morning systolic blood pressure (mmHg)	120.6 (10.7)
CA-IMT (mm)	0.64 [0.50–0.83]
HbA1c (%)	8.6 (1.6)
(mmol/mol)	71.2 (17.4)
Fasting plasma glucose (mg/dL)	122.1 (22.5)
(mmol/L)	6.9 (1.7)
non-HDL- cholesterol (mg/dL)	136.0 (38.9)
(mmol/L)	3.5 (1.0)
eGFR (ml/min per 1.73 m^2^)	81.2 (18.0)
Urine albumin-to-creatinine ratio (mg/g)	8.2 (5.3)
Total sleep time (min)	363.0 (82.6)
REM sleep latency (min)	81.2 (29.5)
REM sleep (min)	84.9 (35.0)
S1,S2 sleep (min)	262.1 (61.9)
Slow wave sleep (min)	2.0 [0.0–24.0]
Apnea hypopnea index	7.1 [2.9–12.5]

Continuous variables are summarized as mean (SD), whereas medians [interquartile range] are shown for variables with skewed distributions. Prevalence was reported as a percentage. BMI = body weight (kg)/height (m^2^). Delta power is the total power of the electroencephalograph signal within the delta frequency bands during REM sleep latency.

CA-IMT, carotid artery intima-media thickness; HbA1c, glycosylated hemoglobin; non-HDL-C, non-high density lipoprotein cholesterol; eGFR, estimated glomerular filtration rate; REM, rapid eye movement.

On average, sleep periods had a mean length of 428.6 min (SD, 82.1) in which participants obtained 363.0 min (SD, 82.6) of total sleep time. This included 262.1 min (SD, 61.9) of S1/S2 sleep, 2.0 min (0.0–24.0 min) of SWS, and 84.9 min (SD, 35.0) of REM sleep. The mean REM sleep latency was 81.2 min (SD, 29.5), which did not differ significantly between those with and without insulin and/or oral hypoglycemic agents (80.3 (SD, 27.2) vs. 85.9 (SD, 40.9) min, p = 0.873), statins (82.5 (SD, 33.9) vs. 80.6 (SD, 27.4) min, p = 0.976), and anti-hypertensive drugs (83.5 (SD, 28.4) vs. 79.7 (SD, 30.4) min, p = 0.292). No significant differences were found in REM sleep latency between those with and without diabetic painful neuropathy (87.0 (SD, 42.0) vs. 80.2 (SD, 27.2) min, p = 0.984).

### Univariate correlations of the clinical variables with sleep architecture

**Fig 1:**
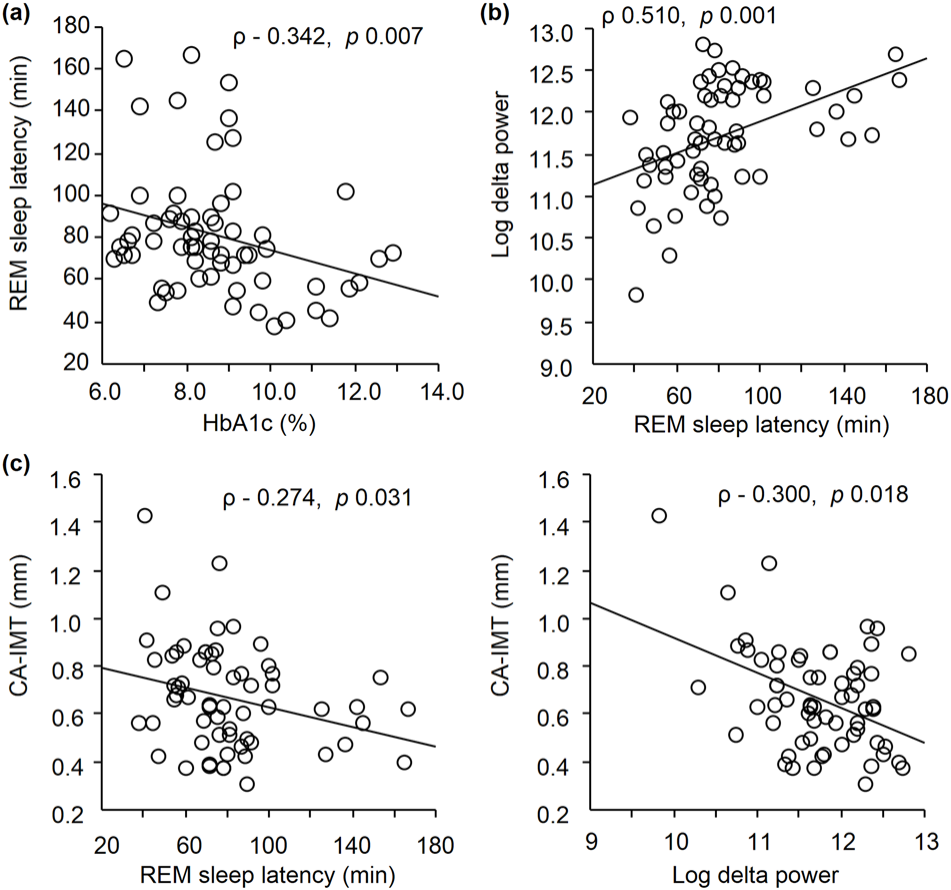
Correlations between HbA1c, REM sleep latency, and log delta power and their effects on CA-IMT. **(a)** HbA1c exhibited significant negative correlation with REM sleep latency (ρ = −0.342, p = 0.007). **(b)** REM sleep latency exhibited significant and positive correlation with log delta power during REM sleep latency (ρ = 0.510, p = 0.001). **(c)** CA**-**IMT exhibited significant and negative correlation with REM sleep latency (ρ = −0.274, p = 0.031) and log delta power during REM sleep latency (ρ = −0.300, p = 0.018). HbA1c, glycosylated hemoglobin; REM, rapid eye movement; CA-IMT, carotid artery intima-media thickness.

The correlations between various clinical variables and total sleep time, REM sleep latency, and the lengths of sleep stages were examined by univariate regression analysis. No significant correlations were observed for markers of glycemic control (i.e. HbA1c and fasting plasma glucose) with total sleep time and the lengths of sleep stages. However, REM sleep latency exhibited a significant and negative correlation with HbA1c (ρ = −0.342, p = 0.007) (Table 2, Fig 1a) and fasting plasma glucose (ρ = −0.292, p = 0.021), and a significant and positive correlation with the AHI (ρ = 0.365, p = 0.004), and tended to positively correlate with BMI (ρ = 0.248, p = 0.051) (Table 2).

**Table 2:** Univariate and Multivariate association of the clinical variables with REM sleep latency and slow wave sleep in patients with type 2 DM.

	REM sleep latency (min)						Slow wave sleep (min)			
Measure	Univariate model		Multivariate model				Univariate model		Multivariate model	
			Model 1		Model 2					
	ρ	p	β	p	β	p	ρ	P	β	p
Gender (0 male, 1 female)	−0.116	0.360					0.113	0.373		
Age (years)	0.019	0.880	0.190	0.182	0.215	0.119	−0.366	0.003*	−0.308	0.047*
DM duration (years)	0.114	0.370					−0.318	0.012*	−0.081	0.591
BMI (Kg/m^2^)	0.248	0.051	0.155	0.314	0.123	0.414	−0.081	0.522		
Morning systolic blood pressure (mmHg)	−0.229	0.078	−0.275	0.042*	−0.297	0.022*	−0.061	0.640		
Apnea hypopnea index	0.365	0.004*	0.031	0.829	0.122	0.390	−0.073	0.570		
HbA1c (%)	−0.342	0.007*	−0.280	0.033*			0.119	0.347		
Fasting plasma glucose (mg/dL)	−0.292	0.021*			−0.349	0.006*	0.160	0.208		
non-HDL- cholesterol (mg/dL)	0.017	0.893					0.084	0.507		
eGFR (ml/min per 1.73 m^2^)	0.023	0.857					0.260	0.040*	−0.040	0.756
Urine albumin-to-creatinine ratio (mg/g)	−0.039	0.756					0.194	0.126		
R^2^	−		0.215		0.259		−		0.123	
p	−		0.024		0.006		−		0.049	

Overall p values were assessed by Spearman rank correlation analysis. BMI = body weight (kg)/height (m^2^). Age and the variables that had a p value of <0.1 by univariate analysis were included in the multivariate regression analysis.

REM, rapid eye movement; HbA1c, glycosylated hemoglobin; non-HDL-C, non-high density lipoprotein cholesterol; eGFR, estimated glomerular filtration rate.

* p< 0.05.

The length of the SWS stage exhibited a significant and negative correlation with age (ρ = −0.366, p = 0.003) and DM duration (ρ = −0.318, p = 0.012), and a significant and positive correlation with eGFR (ρ = 0.260, p = 0.040) (Table 2).

### The variables associated with REM sleep latency and SWS stage length by multivariate analysis

To identify possible factors contributing to decreased REM sleep latency and SWS stage length, a multivariate regression analysis was performed (Table 2). Age and other variables with a p value of <0.10 by univariate analysis were included in the multivariate regression analysis. In model 1, the significant and independent contributor to REM sleep latency was HbA1c (β = −0.280, p = 0.033) and morning systolic blood pressure (β = −0.275, p = 0.042) in the model with age, BMI and AHI as independent variables. In model 2, when HbA1c was replaced with fasting plasma glucose, fasting plasma glucose (β = −0.349, p = 0.006) and morning systolic blood pressure (β = −0.297, p = 0.022) were independently associated with REM sleep latency.

The significant and independent contributor to the length of SWS stage was age (β = −0.308, p = 0.047) in the model with DM duration and eGFR as independent variables (Table 2).

### Characteristics of sleep architecture categorized by tertile of glycemic control

To further explore the relationship of sleep architecture with glycemic control, patients were grouped into 3 categories of glycemic control and were compared using a Kruskal-Wallis test followed by Dunnett's post hoc analysis (Table 3). REM sleep latency decreased with increasing HbA1c with a significant trend (p value for trend is 0.004). Those in the poor glycemic control categories (HbA1c ≥ 9.0% [75 mmol/mol]) had significantly more decreased REM sleep latency (68.5 min (SD, 22.6)) than both suboptimal (HbA1c 7 to 9% [53–75 mmol/mol]) and desirable (HbA1c < 7.0% [53 mmol/mol]) glycemic control categories (suboptimal:87.3 min (SD, 29.3), p<0.05; desirable:94.6 min (SD, 32.7), p < 0.05). Age, total sleep time and the lengths of sleep stages were similar across categories.

**Table 3:** Characteristics of sleep architecture in patients with type 2 DM categorized by HbA1c tertiles.

	HbA1c tertiles			
	< 7.0%	7–9%	≥ 9%	
	(< 53mmol/mol)	(53–75mmol/mol)	(≥ 75mmol/mol)	p for trend
	N = 10	N = 31	N = 22	

Age (years)	57.0 (14.6)	58.4 (14.2)	56.3 (11.2)	0.668
Total sleep time (min)	383.9 (100.6)	361.8 (83.0)	355.0 (75.4)	0.418
REM sleep latency (min)	94.6 (32.7)	87.3 (29.3)	68.5 (22.6) * †	0.004
REM sleep (min)	99.6 (47.2)	83.2 (34.6)	80.6 (28.6)	0.249
S1,S2 sleep (min)	276.1 (71.0)	265.4 (60.2)	251.2 (61.0)	0.573
Slow wave sleep (min)	1.5 [0–24.5]	3.5 [0–17.5]	2.5 [0–30.0]	0.598

Parameters were analyzed by Kruskal-Wallis test followed by Dunnett's post hoc analysis.

REM, rapid eye movement; HbA1c, glycosylated hemoglobin.

*P less than 0.05 as compared with HbA1c < 7%,

^†^as compared with HbA1c 7–9%.

### REM sleep latency and length of SWS stage associated with sleep depth during REM sleep latency by univariate and multivariate analysis

To further confirm the association of REM sleep latency or length of SWS stage with sleep depth during REM sleep latency in patients with type 2 DM, we examined the correlation between total sleep time, REM sleep latency, and lengths of sleep stages with delta power during REM sleep latency, associated with the amount of deep sleep [15]. In univariate regression analysis, REM sleep latency (ρ = 0.510, p = 0.001) (Fig 1b) and length of SWS stage (ρ = 0.574, p = 0.001) exhibited a significant and positive correlation with log delta power during REM sleep latency. Even after multivariate regression analysis including REM sleep latency and length of SWS stage simultaneously as independent variables, either REM sleep latency (β = 0.544, p = 0.001) or length of SWS stage (β = 0.599, p = 0.001) emerged as significant factors and were independently associated with log delta power during REM sleep latency.

No significant correlations were observed with log delta power during REM sleep latency for total sleep time (ρ = 0.140, p = 0.270) and for the lengths of REM sleep stage (ρ = −0.163, p = 0.199), and the S1/S2 sleep stage (ρ = −0.020, p = 0.875).

### Clinical variables associated with CA-IMT by univariate analysis

To examine whether or not a decrease in REM sleep latency and log delta power (during that period) were related to atherosclerosis in patients with type 2 DM, we investigated the correlations between CA-IMT and REM sleep latency or log delta power during that period together with the various traditional risk factors for atherosclerosis (Table 4). Among these, REM sleep latency (ρ = −0.274, p = 0.031) and log delta power (ρ = −0.300, p = 0.018) exhibited significant and negative correlations with CA-IMT (Fig 1c), whereas age (ρ = 0.489, p = 0.001), DM duration (ρ = 0.306, p = 0.015), morning systolic blood pressure (ρ = 0.333, p = 0.010) and HbA1c (ρ = 0.267, p = 0.035) exhibited significant and positive correlations with CA-IMT.

**Table 4:** Univariate correlations of the clinical variable with CA-IMT in patients with type 2 DM.

Measure	CA-IMT (mm)	
	ρ	p
Gender (0 male, 1 female)	−0.123	0.332
Age (years)	0.489	0.001*
DM duration (years)	0.306	0.015*
BMI (Kg/m^2^)	−0.160	0.207
Morning systolic blood pressure (mmHg)	0.333	0.010*
Apnea hypopnea index	−0.122	0.344
HbA1c (%)	0.267	0.035*
Fasting plasma glucose (mg/dL)	0.041	0.745
non-HDL- cholesterol (mg/dL)	0.032	0.798
eGFR (mL/min per 1.73m^2^)	−0.201	0.114
Urine albumin-to-creatinine ratio (mg/g)	0.199	0.116
Total sleep time, min	−0.153	0.227
REM sleep latency, min	−0.274	0.031*
REM sleep, min	−0.042	0.739
S1,S2 sleep, min	−0.141	0.267
Slow wave sleep, min	0.005	0.970
Log delta power	−0.300	0.018*

Overall p values were assessed by Spearman rank correlation analysis. BMI = body weight (kg)/height (m2). Delta power is the total power of the electroencephalograph signal within the delta frequency bands during REM sleep latency.

CA-IMT, carotid artery intima-media thickness; HbA1c, glycosylated hemoglobin; non-HDL-C, non-high density lipoprotein cholesterol; eGFR, estimated glomerular filtration rate; REM, rapid eye movement.

* p< 0.05.

### REM sleep latency and delta power associated with CA-IMT by multivariate analysis

To examine whether or not decreases in REM sleep latency and delta power during the period were independently associated with impaired CA-IMT, multiple regression analysis were performed (Table 5). In model 1, REM sleep latency, HbA1c, and the factors that correlated with CA-IMT (i.e., age, DM duration, and morning systolic blood pressure) were included as independent variables. REM sleep latency (β = −0.232, p = 0.038), age (β = 0.319, p = 0.012), DM duration (β = 0.297, p = 0.022) but not HbA1c and morning systolic blood pressure emerged as a factors independently associated with CA-IMT. In model 2, log delta power replaced REM sleep latency. Log delta power (β = −0.257, p = 0.023) and DM duration (β = 0.274, p = 0.032) were independently associated with CA-IMT.

**Table 5:** Multivariate association of the clinical variable with CA-IMT in patients with type 2 DM.

Measure	Model 1		Model 2	
	β	p	β	p
Age	0.319	0.012*	0.220	0.088
DM duration	0.297	0.022*	0.274	0.032*
Morning systolic blood pressure	0.149	0.178	0.170	0.115
HbA1c	0.190	0.080	0.204	0.054
REM sleep latency	−0.232	0.038*		
Log delta power			−0.257	0.023*
R^2^	0.456		0.465	
p	< 0.001		< 0.001	

Delta power is the total power of the electroencephalograph signal within the delta frequency bands during REM sleep latency.

CA-IMT, carotid artery intima-media thickness; HbA1c, glycosylated hemoglobin; non-HDL-C, eGFR, estimated glomerular filtration rate; REM, rapid eye movement.

* p< 0.05

## Discussion

In the present study, we demonstrated that higher HbA1c was significantly and independently associated with a reduction of REM sleep latency in patients with type 2 DM, suggesting that poor glycemic control was associated with impairment of sleep quality through shortening the most important deepest sleep cycle [23]. Furthermore, impaired sleep quality, as reflected by reduction of REM sleep latency, might be involved in the development of atherosclerosis because of the negative association with CA-IMT. Log delta power (the marker of deep sleep [15]) during REM sleep latency, correlated with both REM sleep latency and SWS stage length; this suggests that log delta power was as an index of deep sleep not only during the first sleep cycle but also during whole sleep cycle. Because log delta power, as well as REM sleep latency, was significantly and independently associated in a negative manner with CA-IMT, it was suggested that the impairment of deep sleep, particularly during the first sleep cycle, might be involved in the development of atherosclerosis in patients with type 2 DM, although causality cannot be determined from the present study.

Our study showed that, among the parameters of sleep time and sleep stages that were studied, REM sleep latency alone had a significant and negative correlation with HbA1c in patients with type 2 DM. Especially, those in the poor glycemic control categories (HbA1c > 9.0% [75 mmol/mol]) had significantly more decreased REM sleep latency than suboptimal and desirable control categories. Reduction of REM sleep latency is known to arise from activation of the hypothalamic–pituitary–adrenal (HPA) axis [24]. Because patients with type 2 DM are reported to exhibit an increase of HPA axis through poor glycemic control [25], this mechanism might be involved in the reduction of REM sleep latency. The decrease in deep sleep might reflect decreased REM sleep latency in patients with type 2 DM, because REM sleep latency correlated in a positive manner with log delta power during REM sleep latency in the present study. This result was in line with the previous findings that among the several sleep cycles, the deepest sleep represented by delta wave activity occurs mostly in REM sleep latency [26]. These data support the notion that deep sleep in REM sleep latency might be a major determinant of sleep quality in type 2 DM patients.

Another disease accompanied with decreased REM sleep latency is depression [27]. Type 2 DM patients in general are known to have higher incidence of depression compared to healthy subjects [28]. Therefore, it is possible that the association between poorer glycemic control and reduced REM sleep latency might be mediated in part by higher incidence of depression in type 2 DM patients.

CA-IMT is established as an early marker of atherosclerosis and predictor of cardiovascular events [10]. In the present study, REM sleep latency and delta power (the maker of deep sleep [15]) during the period, but not HbA1c, exhibited significant and negative correlations with CA-IMT in patients with type 2 DM. Clinical observations suggest that sleep disorders with decreases in deep sleep might serve to elevate nocturnal catecholamine levels [29] and biomarkers of atherosclerosis such as interleukin-6 [30]. We recently showed an association of poor glycemic control with morning blood pressure surge, which has been linked to atherosclerosis [7]. Decrease of REM sleep latency by poor glycemic control may be combined with the process, because REM sleep latency was marginally and negatively correlated with morning systolic blood pressure in the present study. Some prospective studies have elucidated insomnia as a significant predictor for increased risk for complication of atherosclerosis (e.g. coronary heart disease or stroke) [31, 32]. Combined with these findings, it is probable that poor sleep quality might be involved in the development of atherosclerosis in type 2 DM patients.

Taken together, these data suggest that poor glycemic control might impair sleep quality in type 2 DM patients, by which their atherosclerotic change might be accelerated. Although the improvement of glycemic control is assumed to the most effective target to be treated for the protection of atherosclerosis, the treatment of insomnia could be also important site to be intervened for the protection of vascular injury in type 2 DM patients.

In conclusion, our results demonstrate that poor glycemic control is independently associated with impaired sleep quality as represented by decrease of REM sleep latency, and that deterioration of sleep quality might be significantly associated with increased arterial thickening. Therefore, the present study raises the possibility that sleep disturbance might be a major target for the prevention of atherosclerosis in patients with type 2 DM.
